# Development of Liver-on-Chip Integrating a Hydroscaffold Mimicking the Liver’s Extracellular Matrix

**DOI:** 10.3390/bioengineering9090443

**Published:** 2022-09-05

**Authors:** Taha Messelmani, Anne Le Goff, Zied Souguir, Victoria Maes, Méryl Roudaut, Elodie Vandenhaute, Nathalie Maubon, Cécile Legallais, Eric Leclerc, Rachid Jellali

**Affiliations:** 1 CNRS, Biomechanics and Bioengineering, Centre de Recherche Royallieu-CS 60319, Université de Technologie de Compiègne, 60203 Compiègne, France; 2 HCS Pharma, 250 rue Salvador Allende, Biocentre Fleming Bâtiment A, 59120 Loos, France; 3 CNRS IRL 2820, Laboratory for Integrated Micro Mechatronic Systems, Institute of Industrial Science, University of Tokyo, 4-6-1 Komaba, Meguro-ku, Tokyo 153-8505, Japan

**Keywords:** organ-on-chip, liver, extracellular matrix, hydroscaffold, spheroid

## Abstract

The 3Rs guidelines recommend replacing animal testing with alternative models. One of the solutions proposed is organ-on-chip technology in which liver-on-chip is one of the most promising alternatives for drug screening and toxicological assays. The main challenge is to achieve the relevant in vivo-like functionalities of the liver tissue in an optimized cellular microenvironment. Here, we investigated the development of hepatic cells under dynamic conditions inside a 3D hydroscaffold embedded in a microfluidic device. The hydroscaffold is made of hyaluronic acid and composed of liver extracellular matrix components (galactosamine, collagen I/IV) with RGDS (Arg-Gly-Asp-Ser) sites for cell adhesion. The HepG2/C3A cell line was cultured under a flow rate of 10 µL/min for 21 days. After seeding, the cells formed aggregates and proliferated, forming 3D spheroids. The cell viability, functionality, and spheroid integrity were investigated and compared to static cultures. The results showed a 3D aggregate organization of the cells up to large spheroid formations, high viability and albumin production, and an enhancement of HepG2 cell functionalities. Overall, these results highlighted the role of the liver-on-chip model coupled with a hydroscaffold in the enhancement of cell functions and its potential for engineering a relevant liver model for drug screening and disease study.

## 1. Introduction

Drug discovery and development is a long and complex process involving several steps before commercialization. This process, from identifying the potential molecule to its commercialization, takes up to 10 to 15 years and costs approximately 3 to 5 billion dollars of investment [1,2]. Moreover, approximately 90% of drug candidates fail to receive approval by the regulatory authorities, mainly due to their lack of efficacy or toxic effects [3]. Among the key steps in drug development, the preclinical trials stage makes it possible to evaluate biological efficacy and potential safety problems prior to initiating the clinical phase. This stage involves the use of in vitro models followed by extensive animal testing [4]. However, it is estimated that approximately 90% of molecules that successfully pass the preclinical steps fail during clinical trials [2]. Although useful in preclinical tests, animal models have their limitations and fail to mimic complex human biology because of species differences, resulting in poor extrapolation of the results obtained from animal to human [5,6]. In addition, animal experiments pose problems from an ethical and a regulatory viewpoint [5]. Thus, there is now an increasing need to develop relevant in vitro models that can reliably mimic the human response to drugs [7].

The liver is a major organ that plays an essential role in a variety of functions, such as digestion, storage, the production and secretion of plasma, and essentially the detoxification and purification of blood [8]. As the major site of xenobiotic metabolism, the liver is one of the organs most affected by drug-induced toxicity. Drug-induced liver injury (DILI) is a common cause of liver injury and accounts for approximately 50% of cases of acute liver failure in the United States and Western Europe [9,10,11]. DILI is also the most common cause of a drug’s withdrawal from the market and restriction of use [12]. The failure to detect DILI during the drug development process is attributable to the poor predictability of the screening methods (in vitro, in vivo, ex vivo, and in silico) used in the preclinical phase [9,11,13,14].

In current in vitro preclinical assays, the hepatotoxicity of drug candidates is most commonly tested using two-dimensional (2D) monolayer cultures [8]. These cultures are mainly performed in a static macroscale environment such as Petri dishes or multi-well plates. Although 2D static cultures have provided significant contributions to biomedical research and the pharmaceutical industry, they fail to both reproduce in vivo physiology and metabolism, and accurately predict cellular responses to drugs [15,16]. These limitations are associated with the lack of specific architecture in the tissues, mechanical and biomechanical cues, and cell–cell and cell-matrix interactions [17]. Several studies have shown that primary human hepatocytes (PHH), which are considered the gold standard for in vitro drug screening, de-differentiate and rapidly lose their key phenotypic and specific detoxification functions, when cultured in 2D static conditions [16,18]. Therefore, there is an increasing need for the development of reliable in vitro human liver models. These models must reproduce as closely as possible the in vivo characteristics of the liver microenvironment.

In an attempt to improve in vitro liver models, different approaches based on tissue engineering, microfabrication, and microfluidics have been proposed during the last decade: 3D cell culture (spheroids, culture in hydroscaffold/hydrogel, and 3D bioprinting), organoids derived from stem cells, dynamic organ-on-chip (OoC) culture, coculture models of different liver cells and liver coculture with other organs [17,19,20]. Of those models, dynamic organ-on-chip and 3D spheroids seem to be two of the most promising models for hepatic cell cultures [21]. In particular, OoC technology makes it possible to build a well-controlled microenvironment and create “physiological-like” situations, such as 3D architectures, cell–cell and cell-matrix interactions, continuous nutrient exchange, zonation, physiological shear stress, and chemical gradients [15,22]. Moreover, microfluidic OoC offers the possibility of reproducing physiological organ-to-organ interactions, when cells from different organs are cultivated in separate biochips and chemical factor exchange is made possible through microfluidic tubing [23]. The culture in 3D spheroids also exhibits several features making it possible to both mimic in vivo cell conditions and maintain liver-specific functions. It promotes adhesion between cells, their interaction with the extracellular matrix (ECM), and the development of gas, nutrients, and metabolite gradients [17,24,25]. Spheroids can be produced by self-aggregation of cells (non-adhesive surface, bioreactor, hanging drop technique) or using a hydrogel/scaffold matrix [25,26]. The use of hydrogel and scaffold offers the possibility of tuning the cell microenvironment by modifying the composition of the matrix and/or the mechanical properties [17,25].

In previous works, our group has developed liver-on-chip models with different hepatic cells (HepG2/C3A, HepaRG, PHH, primary rat hepatocytes and human induced pluripotent stem cells hiPSCs) to investigate liver metabolism [27,28], drugs and pesticide toxicity [29,30,31], and liver regeneration and the development process [32]. Recently, we integrated an alginate cryogel into our biochip to promote 3D cell organization. The cells colonize the entire surface of the collagen-coated cryogel, forming a thick (200 µm) tissue-like 3D structure from the bottom to the top of the biochip [33]. In the present study, we propose a new liver-on-chip model integrating a hydroscaffold allowing cells to organize into a complex 3D spheroid architecture. To promote a more in vivo-like environment for cells, we used a hydroscaffold containing the key liver extracellular matrix (ECM) components (hyaluronic acid (HA), RGDS, galactosamine, collagen I and IV). We studied the behavior and functionalities of HepG2/C3A, a liver cell line often used as an in vitro model for human hepatocytes, cultured in dynamic conditions in the biochip integrating the scaffold. Different cell densities and times of culture, ranging from 4 days (short-term culture) to 21 days (long-term culture), were investigated.

## 2. Materials and Methods

### 2.1. Biochip Fabrication

The details of the biochip design and manufacturing process have been reported in our previous works (Figure S1) [34,35]. The biochip consists of a cell culture chamber with a volume of 40 μL and a 2 cm^2^ surface area of cell growth. The culture chamber is the result of assembling two polydimethylsiloxane layers (PDMS, Sylgard 184 kit, Dow Corning, Midland, TX, USA) manufactured by soft lithography. The microstructures in the bottom layer define the cell culture chambers and microchannels with a depth of 100 µm. The top layer, with a reservoir 100 µm in depth, includes an inlet and an outlet for culture medium perfusion. The surfaces of the two PDMS layers were oxidated by reactive air plasma (1 min at 30 W, Harrick Scientific, Ithaca, NY, USA) and assembled to form an irreversibly sealed biochip. Two polypropylene connectors (female Luer ID = 0.16 cm, Cole Parmer, Vernon Hills, IL, USA) were introduced into the inlet and outlet of the biochip. They make it possible to connect the biochip to a syringe or to the perfusion circuit.

### 2.2. Hydroscaffold Preparation

We used an HA-based hydroscaffold developed by HCS Pharma (Loos, France) and called BIOMIMESYS^®^ Liver. It is mainly composed of RGDS-grafted hyaluronic acid (HA-g-RGDS), galactosamine-grafted hyaluronic acid (HA-g-GalN), and extracellular matrix (ECM) proteins (collagen type I and collagen type IV). The hydroscaffold crosslinking is performed using adipic acid dihydrazide crosslinker (ADH).

The hydroscaffold was integrated into the biochip (described in Section 2.1) by HCS Pharma using the previously patented process [36]. Briefly, the pseudo-hydrogel (HA-g-RGDS, HA-g-GalN, collagen and ADH) was injected into the biochip and the hydroscaffold transformation was performed in situ. Then, washing, freeze-drying, and UV irradiation steps were performed to make it possible to preserve it until use. For Petri 3D static cultures, BIOMIMESYS^®^ Liver is provided by HCS Pharma in a ready-to-use 48-well plate.

### 2.3. IDCCM Fluidic Platform

The Integrated Dynamic Cell Cultures in Microsystems (IDCCM, developed by our laboratory) is a polycarbonate fluidic platform used to ensure the dynamic culture of cells cultured in the biochip (Figure S2) [28,34]. It is an easy plug-and-play platform making possible flow control through 12 biochips in independent parallel closed loops. The IDCCM device is composed of 3 parts: the cover, composed of connectors to link the device to the peristaltic pump by tubing a PDMS layer for tightness and a bottom layer composed of culture medium reservoirs. The biochips are connected at the bottom of the IDCCM device, and the 3 parts are sealed using a clipper system.

### 2.4. Cell Culture

The HepG2/C3A, a clone of the HepG2 line derived from human hepatocellular carcinoma (ATCC CRL-10741), was used as a liver cell model for experiments. HepG2/C3A cells were cultured in Minimal Essential Medium (MEM) with phenol red (Pan Biotech, Aidenbach, Germany) supplemented with 10% (*v*/*v*) fetal bovine serum (Gibco, Waltham, MA, USA), 2 mM L-glutamine (Gibco), 0.1 mM non-essential amino acids (Gibco), 1 mM sodium pyruvate (Gibco), and 100 U/mL/100 μg/mL penicillin/streptomycin (Pan Biotech). For maintenance, the cells were cultured in 75 cm^2^ flasks at 37 °C in a humidified atmosphere supplied with 5% of CO_2_. The cells were passaged weekly at a confluence of 80–90% and the culture medium was renewed every two days.

**Figure 1:**
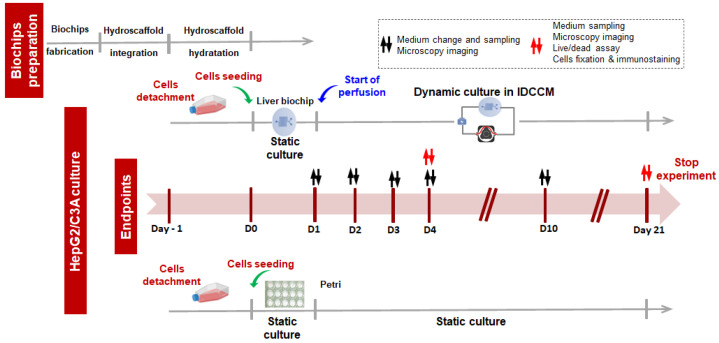
Experimental procedures used for HepG2/C3A cell culture in the biochip and Petri containing the HA-hydroscaffold.

Before cell seeding, the hydroscaffolds in the biochips were hydrated with culture medium. Then, the cells, between passages 12 and 23, were detached from the 75 cm^2^ flasks using trypsin-EDTA (0.25%, Gibco), counted, and the appropriate number of cells was seeded into the biochips. After 24 h (37 °C, 5% CO_2_) of static conditions, the biochips were connected to the IDCCM device, and the perfusion was started using a peristaltic pump (10 µL/min). The whole system (pump and IDCCM device containing biochips) was placed in an incubator at 37 °C and 5% of CO_2_. The detailed experimental procedure is shown in Figure 1. The same number of cells was seeded directly into the wells of a 48-well plate containing the hydrated hydroscaffold (static control).

### 2.5. Cell Viability

The LIVE/DEAD^TM^ kit (Thermo Fisher Scientific, Waltham, MA, USA) was used to stain the cells. Ethidium homodimer-1 (excitation peak at 528 nm and emission peak at 617 nm) was used at a concentration of 4 μM to stain the dead cells, while calcein-AM (excitation peak at 495 nm and emission peak at 520 nm) was used at 2 μM to stain the living cells. Viability was assessed at the end of the experiment. Briefly, after washing with phosphate-buffered saline (PBS, Gibco), a solution of culture medium containing ethidium/calcein was added and the biochips were incubated at 37 °C, 5% CO_2_. After 20 min, the biochips were rinsed with PBS and observed using a conventional fluorescence microscope (Leica DMI 6000B, Leica Microsystems, Wetzlar, Germany).

### 2.6. Immunostaining Assays

Immunofluorescence observations were performed at the end of the experiments. We selected to stain F-actin (morphology of the actin cytoskeleton), E-cadherin (mediator for cell–cell adhesion), MRP2, and BSEP (cell polarity and bile canalicular network development).

At the end of the perfusion, the biochips were washed with PBS, fixed in paraformaldehyde 4% (PFA, MP Biomedicals, Illkirch, France) for 30 min at room temperature and washed and stored in PBS until staining. Before staining, the cells were permeabilized with 0.5% Triton X-100 solution for 30 min and blocked with a 1% bovine serum albumin solution in PBS (BSA, Sigma Aldrich, St. Louis, MO, USA) for 30 min. The antibodies used were mouse anti-E-cadherin (BDB610181, BD Biosciences, San Jose, CA, USA), rabbit anti-MRP2 (M8316, Sigma Aldrich), rabbit anti-ABCB11/BSEP (ab155421, Abcam, Cambridge, UK), donkey anti-mouse Alexa Fluor 647 (ab150107, Abcam), goat anti-rabbit Alexa Fluor 680 (A21109, Invitrogen, Waltham, MA, USA), and goat anti-rabbit Alexa Fluor 488 (A11034, Invitrogen). All antibodies were diluted in the range recommended by the manufacturers. Primary and secondary antibodies were incubated overnight at 4 °C in the dark. Cell nuclei were stained with DAPI at 10 µg/mL (4′,6-diamidino-2-phenylindole, D1306, Invitrogen), and phalloidin (Alexa Fluo 488 Phalloidin, Thermo Fisher) staining was used for F-actin visualization. The observations of the stained samples were made with a laser scanning confocal microscope (SM 710, Zeiss, Oberkochen, Germany).

### 2.7. Albumin and Urea Measurements

ELISA sandwich assay was used to quantify the albumin concentration in the culture media collected throughout the experiments (dynamic biochips and static multi-well plates). The assays were performed using a human albumin ELISA Quantitation Set (E80-129, Bethyl Laboratories, Montgomery, TX, USA), following the manufacturer instructions.

For urea quantification in the culture medium, a colorimetric method was used (urea assay kit, QuantiChrom DIUR-100, BioAssay Systems, Hayward, CA, USA). The kit contains a chromogenic reagent that forms a colored complex specifically with urea. For both assays, the results were obtained with a Spectafluor Plus microplate reader (TECAN, Männedorf, Switzerland) set to a wavelength of 450 and 520 nm for albumin and urea, respectively.

### 2.8. Flow Control and Pressure Drop Measurement

Following integration of the hydroscaffold, and at different steps in the cell culture (time points), the biochips were connected to a pressure-controlled pumping system. The circuit was composed of a pressure controller (MFCS-EX, Fluigent, Le Kremlin-Bicêtre, France) connected to the biochip and to a flowmeter (Flow Unit type M, Fluigent). The tubes containing the culture medium were pressurized to deliver a flow into the microfluidic installation. Downstream of the biochip, medium flowed through the flowmeter. The pressure applied to the inlet reservoir was adjusted through a feedback loop to maintain the desired target flow rate. The entire setup is presented in Figure S3.

The pressure drop (pressure difference between inlet and outlet reservoirs) was measured at different flow rates (from 0 to 30 µL/min) for the empty biochip as a reference and compared with the biochip containing the hydroscaffold. Then, real time monitoring was performed for a flow rate of 10 µL/min throughout the cell culture experiments.

### 2.9. Scanning Electron Microscopy (SEM)

SEM analysis was performed with samples fixed in paraformaldehyde 4%. The biochips were frozen in liquid nitrogen to solidify the PDMS and thin slices were cut to observe the organization and distribution of the cells and hydroscaffold matrix inside the culture chambers. The images were taken using an XL30-ESEM FEG (Philips, Eindhoven, The Netherlands).

### 2.10. Statistical Analysis

All experiments were repeated at least three times and a minimum of 2 biochips/conditions were used in each experiment (*N* = 3 experiments and 6 ≤ *n* (biochip) ≤ 12). The data are presented as the mean ± standard deviations (SD). To determine significant statistical differences, a one-way ANOVA test was performed using GraphPad Prism 8.4.3 software (GraphPad, San Diego, CA, USA). Data with *p*-values < 0.05 were identified as statistically significant and highlighted with an asterisk in the figures.

## 3. Results

### 3.1. Integration of the Hydroscaffold into the Biochip

**Figure 2:**
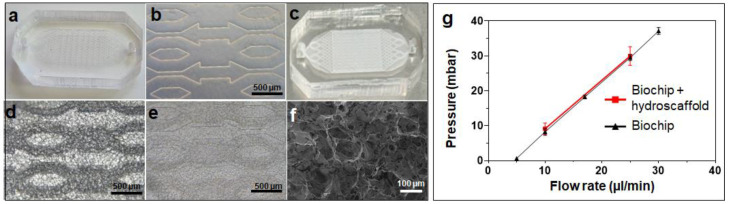
Microfluidic devices and hydroscaffold characterization. (**a**) microfluidic biochip; (**b**) microscopic observation of biochip microstructures; (**c**) biochip containing the hydroscaffold; (**d**) microscopic observation of dehydrated hydroscaffold inside biochip; (**e**) microscopic observation of hydrated hydroscaffold inside biochip; (**f**) SEM observation of the hydroscaffold; and (**g**) characterization of the pressure variation in the biochip with and without hydroscaffold.

The hydroscaffold crosslinking was performed in situ in the biochip. The purified pseudo-hydrogel was easily injected into the biochip using a syringe and the hydroscaffold transformation reaction took place for 2 h. Figure 2a–e shows the pictures and optical microscope observations of the biochips without and with the hydroscaffold, respectively. The hydroscaffold is easily identifiable both in the picture (Figure 2c, white color) and in the microscopic observations (Figure 2d,e, dried and hydrated hydroscaffold, respectively). It is well distributed and homogenously occupies the entire space of the biochip, from the inlet to the outlet. The contrast in color observed in Figure 2d is due to the difference in height between the bottom of the biochip and the top of the microstructures. The SEM observations of the scaffold highlighted a homogeneous porous network with a pore size of approximately 120 ± 20 µm (Figure 2f).

Considering the possible additional resistance to flow generated by the scaffold integration, it was important to confirm that culture medium can circulate and is distributed evenly inside the biochip. To investigate this effect, biochips with and without a hydroscaffold were connected to a pressure-controlled circuit and the pressure drop was monitored for flow rates relevant for cell culture [37]. The pressure variations plotted against the flow rates are presented in Figure 2g. We found that the two plots (biochips with and without a hydroscaffold) fit well, with no significant difference. Using the equation for hydraulic resistance (R_h_ = ΔP/Q, were R_h_ is the hydraulic resistance, ΔP the pressure variation between the inlet and the outlet of the biochip, and Q the flow rate), we calculated hydraulic resistance of 5.9E + 12 ± 0.4E + 12 and 5.9E + 12 ± 1.0E + 12 kg·m^−4^·s^−1^ for the biochips without and with a hydroscaffold, respectively.

### 3.2. Cell Culture in Biochip Containing the Hydroscaffold

#### 3.2.1. Effect of Cell Seeding Density: Morphology

**Figure 3:**
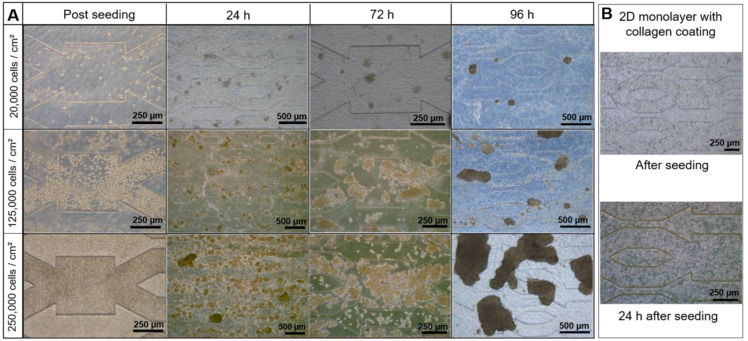
Morphology of HepG2/C3A cells cultivated in (**A**) biochip containing the hydroscaffold and (**B**) biochip coated with collagen.

To evaluate the effect of starting cell density on the formation, size, and organization of spheroids, we investigated three different seeding densities: 20,000 (low density), 125,000 (intermediate density), and 250,000 cells/cm² (high density). For comparison, the high and intermediate densities were chosen based on our previous works with a scaffold-free biochip and biochip containing alginate cryogel [33,37]. The evolution in cell morphologies throughout 96 h of culture in biochips containing a hydroscaffold (including 24 h of adhesion and 72 h of dynamic culture) are illustrated in Figure 3A.

24 h after seeding, the cells embedded in the hydroscaffold started to aggregate and create spheroids. The number and size of the spheroids were proportional to the starting cell density. Furthermore, spheroids created from high and intermediate densities were less uniform in size and shape, compared to spheroids resulting from low seeding density. In comparison, 24 h after seeding in a scaffold-free biochip, the cells adhered to the bottom of the biochip and formed a monolayer (Figure 3B). After the medium flow started, the spheroids remained embedded in the scaffold and grew continuously, especially for high and intermediate starting densities. After 96 h of culture, they created irregular large spheroids or cell aggregates (Figure 3A). In the case of low density seeding, we obtained uniform spheroids with an approximate diameter of 100–200 µm (Figure 3A). From 8–10 days of culture with high and intermediate seeding densities, the growth of spheroids led to the formation of large clusters of spheroids in the whole biochip. These clusters blocked the flow as indicated by a significant increase in the pressure drop (detailed in Section 3.3), which played a part in damaging the perfusion circuit (results not shown). Finally, the cultures in a well-plate containing a hydroscaffold showed similar results after 96 h of culture: large spheroids/aggregates with high and intermediate density and uniform spheroids in the case of the low starting density (Figure S4).

#### 3.2.2. Cell Viability and Functionality

**Figure 4:**
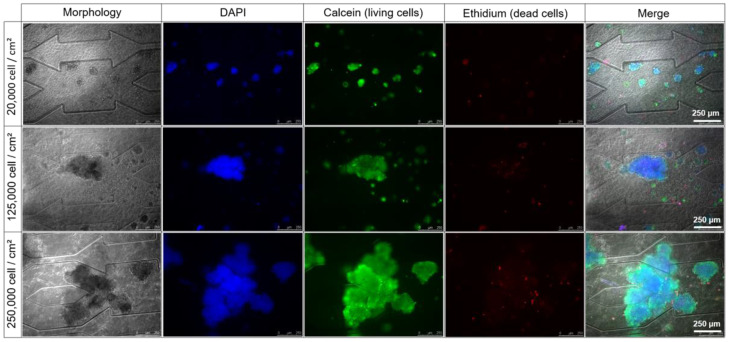
Cell viability for different seeding densities after 96h of culture in the biochip containing the hydroscaffold: DAPI (nuclei, blue), calcein (living cells, green), and ethidium (dead cells, red).

After 96 h of culture (24 h in static and 72 h in dynamic conditions), cell viability was evaluated with live/dead staining for the three starting densities. As shown in Figure 4, all spheroids presented uniform green fluorescent intensity (living cells). The red fluorescent signal was very low, indicating a negligible number of dead cells in the three conditions in comparison with the number of living cells. However, the red fluorescence intensity in the spheroids obtained from high and intermediate starting densities seemed to be higher compared to spheroids created with low density.

**Figure 5:**
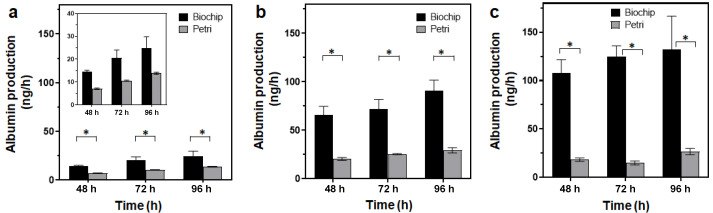
Albumin secretion by HepG2/C3A cultivated in a dynamic biochip and static Petri containing the hydroscaffold. Starting cell density 20,000 (**a**), 125,000 (**b**), and 250,000 cells/cm² (**c**); * *p* < 0.05 The insert in panel (**a**) is a close-up on the vertical axis.

To evaluate the effect of culture in a biochip containing a scaffold on HepG2/C3A specific functions, albumin production was quantified and compared, with results obtained in static culture in a well-plate with a hydroscaffold. The results are shown in Figure 5. In both culture modes (dynamic and static), albumin production increased from day 2 to day 4 for the three starting densities. However, albumin production in biochips containing a scaffold were approximately 2, 3, and 10-fold higher than the 3D hydroscaffold in Petri for low, intermediate, and high starting densities, respectively. In the biochip, albumin production after 4 days of culture reached 25 ± 8 ng/h for low starting density (Figure 5a), 91 ± 18 ng/h for intermediate starting density (Figure 5b), and 132 ± 34 ng/h for high starting density (Figure 5c).

Table 1 summarizes albumin production in the biochip containing a hydroscaffold and in two other biochips from our previous works: a biochip containing alginate cryogel and a hydroscaffold/cryogel-free biochip [33,37]. For the starting density of 250,000 cells/cm^2^, there was no significant difference between the three types of biochips. At 96 h, albumin production was 132 ± 34 ng/h in the biochip with the hydroscaffold, 135 ± 60 ng/h in the biochip with alginate, and 190 ± 85 ng/h in the empty biochip. Similar albumin production was also found with a starting density of 125,000 cells/cm^2^ in the biochip containing a hydroscaffold and the empty biochip.

### 3.3. Long-Term Cell Culture in a Biochip Containing the Hydroscaffold

The longevity of in vitro liver models is a critical parameter. Hepatotoxicity most often manifests after a long time and several exposures to drugs. To evaluate our model in long-term culture, and considering the results obtained in the previous section, we chose to work with a low starting density (20,000 cells/cm^2^) to prevent the formation of large clusters of spheroids and create flow blockage.

#### 3.3.1. Cell Proliferation and Spheroid Formation

A total of 20,000 HepG2/C3A cells/cm^2^ (40,000 cells/biochip) were seeded into the biochip containing a hydroscaffold. After 24 h in static conditions, the pump was started at 10 µL/min. The flow rate was maintained constant throughout the experiment and the pressure was monitored using the setup described in Section 2.8.

**Figure 6:**
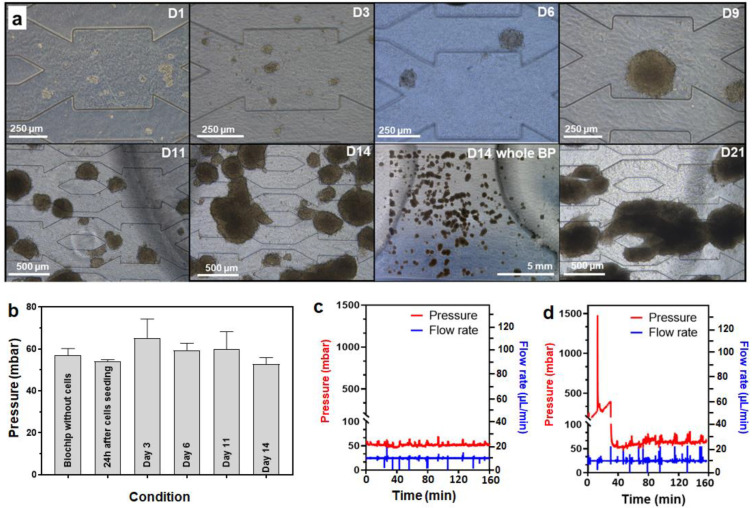
Long-term (21 days) culture of HepG2/C3A cells in a biochip with a hydroscaffold (Starting cell density of 20,000 cells/cm²). (**a**) evolution in the morphology of HepG2/C3A spheroids throughout the 21 days of culture; (**b**) pressure evolution during the first 14 days of culture; (**c**,**d**) pressure measured inside the biochip on day 14 and 21, respectively.

The evolution of HepG2/C3A spheroids throughout 21 days of culture is presented in Figure 6a. From day 1, the cells attached to the hydroscaffold and self-aggregated in small clusters of cells. Then, the cell clusters gradually formed spheroids with well-defined shapes throughout the first 11 days of culture. The size of the spheroids increased over time to reach diameters of between 150 and 450 µm by day 11. The pressure inside the biochips remained stable, close to 60 mbar, during this period (first 11 days of culture, Figure 6b). From the 14th day of culture, the cells proliferated strongly, and the spheroids started to overlap and occupy most of the biochip area (Figure 6a). Nevertheless, as shown in Figure 6c, the pressure inside the biochip did not increase. Finally, on day 21 of culture, large clusters of spheroids were formed in the whole biochip (Figure 6a). Consequently, the hydraulic resistance of the biochip increased, affecting the circulation of the culture medium. This was confirmed by a significant pressure jump, reaching 1.5 bar (Figure 6d, the initial pressure at 10 µL/min was 60 mbar). Normal pressure (60 mbar) was restored after detaching a spheroid cluster (nb: the same behavior was observed in Section 3.2.1 with a higher density inoculation but at an earlier time point due to tissue growth).

#### 3.3.2. Spheroid Morphology and Integrity

**Figure 7:**
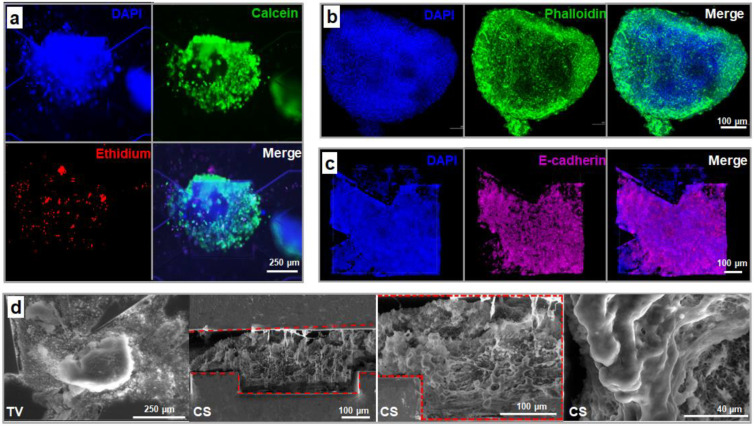
Characterization of HepG2/C3A spheroids after 21 days of dynamic culture in a biochip containing a hydroscaffold. (**a**) cell viability: DAPI (nuclei, blue), calcein (living cells, green) and ethidium (dead cells, red); (**b**) F-actin staining: DAPI (nuclei, blue) and phalloidin (F-actin, green); (**c**) E-cadherin staining: DAPI (nuclei, blue) and E-cadherin (purple); (**d**) SEM observation (TV: top view and CS: cross section). The immunostaining images (**b**,**c**) correspond to z-stack projections.

Live/dead assays were performed on the spheroids at the end of the experiments, after the 21 days of dynamic culture (Figure 7a and Figure S5). Despite the large spheroid size (diameter ≥ 500 µm), the fluorescence images demonstrated high viability (green fluorescent signal) of the cells in the 3D structure. We did not observe any specific necrotic core within the spheroids. Only some dead cells (red fluorescent signal) were observed, distributed over different areas of the spheroids.

The internal structure of the spheroids and cell–cell adhesion and interaction were analyzed using immunofluorescence with phalloidin for F-actin staining and anti-E-cadherin antibody. F-actin plays an important role in the mediation of cell shape and, spreading. As shown in Figure 7b, the actin cytoskeleton of the cells can be seen clearly (intense green fluorescence signal) in the whole spheroid. The actin filaments appeared well organized, creating a complex network throughout the entirety of the spheroid. In parallel, abundant E-cadherin expression was observed in the 3D spheroids, as shown by the purple fluorescence in Figure 7c. The positive staining of E-cadherin confirmed the well-developed adherent junctions, the overall cell adhesion integrity within the spheroids and the epithelial status of the tissue. In comparison, F-actin and E-cadherin networks seemed to be less developed in the spheroids after 21 days of culture in a static well-plate containing a hydroscaffold (Figure S6).

To study the structure and organization of the cells/spheroids inside the biochip containing a hydroscaffold, the samples (fixed after 21 days of culture) were observed under scanning electron microscope (SEM). SEM imaging was performed on the device’s cross-sections and the bottom layer of the biochip (top view) after disbanding the top layer (Figure 7d and Figure S7). The SEM images in the top view show the formation of large 3D spheroids surrounded by the hydroscaffold. The cross-section images confirmed the cell organization in a tissue-like 3D structure from the bottom to the top of the biochips and the cell–scaffold interactions.

#### 3.3.3. Spheroid Functionality

**Figure 8:**
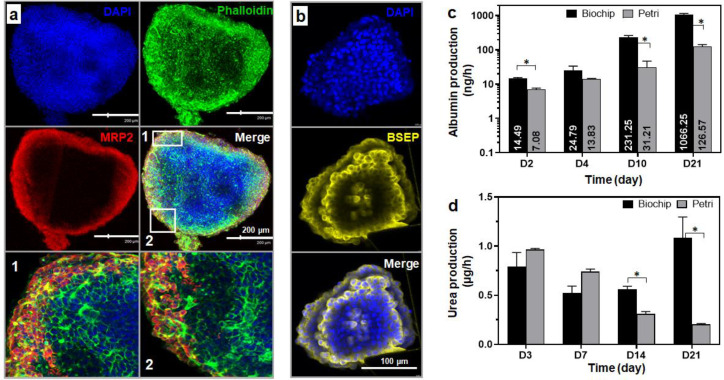
Characterization of HepG2/C3A spheroids after 21 days of dynamic culture in a biochip containing the hydroscaffold. (**a**) F-actin and MRP2 staining showing the formation of bile canalicular-like structures: DAPI (nuclei, blue), phalloidin (F-actin, green); MRP2 (red) and biliary-like network (co-localization MRP2 and F-actin signals, yellow overlay signal, the two pictures in the bottom correspond to an enlargement from the merge picture); (**b**) BSEP staining: DAPI (nuclei, blue) and BSEP (yellow); (**c**,**d**) albumin and urea production in the dynamic biochip and static Petri conditions throughout 21 days of culture (both biochip and Petri contained the hydroscaffold). The immunostaining images (**a**,**b**) correspond to z-stack projections. * *p* < 0.05.

Cell polarization is one of the key characteristics of hepatocytes. To investigate the polarity of the cells within the spheroids and confirm the formation of bile canalicular-like structures, MPR2 (coupled to actin) and BSEP stainings, performed on spheroids after 21 days of culture, were used as markers. BSEP and MRP2 are two proteins localized at the canalicular membrane of the hepatocytes and they normally transport bile acids and drugs from hepatocytes to the bile network. Both stainings confirmed the presence of a biliary-like network within the spheroid. As shown in Figure 8a, the immunofluorescence images highlighted the co-localization of the canalicular MRP2 transporter signal (red) with the actin fluorescent signal (green) resulting in the yellow overlay signal (merge panel in Figure 8a). The expression of the BSEP transporter was demonstrated by the intense yellow fluorescence in Figure 8b.

Albumin production is one of the main functions of the liver and is often used as a specific marker to evaluate hepatocyte functionality. The albumin production from spheroids cultured in dynamic biochips containing a hydroscaffold was quantified over the 21 days of culture and compared to albumin produced in static cultures in a well-plate with a hydroscaffold (Figure 8c). In the biochips, albumin gradually increased throughout the 21 days of the experiment. The productions were of 14.49 ± 1.15, 24.79 ± 8.7, 231.25 ± 30, and 1066.25 ± 83 ng/h at days 2, 4, 10, and 21, respectively. Concerning the static Petri culture, the production of albumin also increased over the 21 days of culture. However, albumin levels were significantly lower than the values obtained in the biochip cultures: 2- and 10-times lower at the beginning (days 2 and 4) and the end (days 10 and 21) of cultures, respectively (Figure 8c).

Finally, urea production was analyzed at different time points during the 21 days of culture (in biochips and Petri cultures, Figure 8d). In biochips, urea synthesis remained at a steady approximate level over the 21 days (between 0.55 ± 0.1 and 1.08 ± 0.36 µg/h). On the other hand, in the static Petri cultures, urea production gradually decreased over time. Productions were of 0.96 ± 0.02, 0.74 ± 0.04, 0.30 ± 0.03, and 0.20 ± 0.01 µg/h at days 3, 7, 14, and 21, respectively.

## 4. Discussion

The literature reports improved liver functions, cellular morphology reorganization, and higher metabolic capability in in vitro models thanks to the integration of advanced bioengineering and biomaterial techniques. Among them, the liver cultures in 3D configurations (spheroids/organoids), coupled or not with the extracellular matrix microenvironment reproduction (due to functionalized gels and hydroscaffolds), have played a part in enhancing hepatic functions [38,39,40,41]. In addition, it has been widely reported that liver cell cultures under flow reproduce zonation-like patterns and reduce the accumulation of waste and toxic compounds [42,43,44,45,46]. Furthermore, coupling both flow and 3D cultures appeared to promote higher physiological relevance, when compared to 3D static cultures [47]. The present work combines the advantages of (i) the 3D configuration cultures using a hydroscaffold mimicking the liver’s extracellular matrix, (ii) dynamic cultures and (iii) PDMS organ on chip (transparent material, gas permeable). The HepG2/C3A cells attached on to the scaffold and proliferated, creating spheroids. Over the culture time, the spheroids became increasingly larger. Spheroids in the perfusion cultures reached a larger size than spheroids in static controls. One specific benefit of the present hydroscaffold relied on its composition, that is, a finely-tuned hyaluronic acid scaffold including RGDS peptide (Arg-Gly-Asp-Ser), galactosamine, collagen type I, and collagen type IV. The choice of extracellular matrix has its importance in epithelial polarization including hepatocytes and liver tissues [48]. The ECM is reported as a key regulator for improving hepatic functionality [49] and liver regeneration [50] but also regulating the development of liver disorders [51]. Furthermore, a complex ECM in hydrogel such as that obtained from the decellularized liver confirmed the importance of this environment in an in vitro model and in in vivo transplantation applications [52]. The BIOMIMESYS^®^ Liver HA-hydroscaffolds’ ECM proteins were selected according to the liver matrix microenvironment [53]. More particularly, previous works have shown that the BIOMIMESYS^®^ Liver in static Petri made it possible to develop a protocol for differentiating human induced pluripotent stem cells (iPSCs) into human liver organoids (including not only hepatocytes but also biliary, stellate, and endothelial cell types) suitable for molecular screening (patent pending). Furthermore, the manufacturer’s own data reported a higher expression of albumin secretion in their 3D Petri culture, when compared to 2D Petri culture of HepG2 cells (Figure S8).

The present study demonstrated that the hydroscaffold allowed higher levels of albumin production in dynamic culture, when compared to static 3D Petri. However, we did not detect any specific difference in albumin secretion with our other biochip technologies (PDMS biochip without gel [34,37] and 3D alginate cryogel biochip [33]). We also confirmed that F-actin and E-Cadherin were significantly organized at the cell–cell contact throughout the spheroids within the biochip containing the HA-hydroscaffold. These observations indicated intercellular adhesive interaction in our tissues that was consistent with the literature [54]. Furthermore, we detected successful polarization of the tissue within the hydroscaffold, which is a typical expectation for HepG2 cell cultures in a 3D configuration and under perfusion in a biochip environment [38,55].

The hydroscaffold integrated into the microfluidic biochip contributed to creating large spheroids, when compared to BIOMIMESYS^®^ Liver 3D Petri. The HepG2/C3A, a liver cell line with a high capacity for proliferation, led to a specific range of uses in the present configuration. The spheroids’ over-growth contributed to clogging the biochips and to blocking fluid flow, which in turn led to the devices’ failure. This was characterized by the fluid leakage that resulted from a significant increase in pressure in the perfusion circuit. This behavior was time-dependent, based on cell inoculation density; lower was the inoculation density, later was the device failure. This phenomenon was also previously observed with our alginate cryogel biochip with HepG2/C3A [56]. Conversely, we never observed this situation in the biochips without gel in which cultures of up to 4 weeks were successful with HepG2/C3A (Figure S9) [57]. In fact, without gel, the cells grow layer-by-layer and their over-growth is limited by the height of the microstructure inside the biochips. Nevertheless, the hydroscaffold biochips made it possible to increase the cell culture density within the biochips, creating a full-scale 3D tissue, concomitantly with a healthy culture given low necrotic cores were observed. This was attributed to better nutrient and oxygen distributions to the spheroids due to their random location within the biochips. Our observations suggested the necessity for a fine balance between the choice of applications (chronic vs. acute biological processes), time of culture, cell density, cell viability, and the type of 3D biochips. Furthermore, it also demonstrated the importance of biochip design and flow perfusion conditions. Additional endothelial cultures may provide an alternative solution via the formation of tubular-like tissues within the hydrogel and spheroids, thus facilitating the circulation of fluid [58,59,60]. Finally, cells with a limited proliferation rate, such as primary hepatocytes, will only mildly modify flow resistance and thus be fully compatible with those technologies, as already demonstrated in our previous works (in L. Boulais, 2020 for alginate cryogel, and by Jellali et al., 2016 for a 3D biochip without gel) [28,56].

Reconstructing an in vitro liver model that mimics in vivo conditions is very challenging and aims to maintain the morphological characteristics and cellular functions of hepatocytes over long periods of culture. In past decades, different liver models based on one or more advanced technologies, such as OoC, 3D spheroids and cells embedded in hydroscaffolds, have been developed [17,20,61]. However, to our knowledge, only a few studies have focused on integrating a hydrogel/hydroscaffold into the biochip and there is no system describing a dynamic liver-on-chip model making hepatocyte cultures possible in spheroids, embedded into a hydroscaffold that closely mimics the liver’s ECM. In this proof of concept, we used HepG2/C3A cells, which are a good compromise between the ease of use and the expression of certain funtions of liver cells [62]. Although PHHs are considered the gold standard for liver models, their use is not suitable for this first stage of development (high costs, complexity of culturing). Overall, our liver-on-chip model made possible the culture of HepG2/C3A cells in 3D spheroids enbedded into liver-like ECM under dynamic flow for a long period (21 days). The cells showed high viability and stable hepatic functions throughout the 21 days of culture. However, the use of other cell models, such as PHHs and hiPSCs-derived hepatocytes, is required to confirm the potential of the device.

In OoC technology, the small amount of cells and culture medium volume represent a major limitation for biological characterization [63]. In our device, a significant number of cells (up to 2–3 million) can be hosted, and each device is perfused with 4 mL of culture medium. Thus, various analyses could be performed with the available biological material. Furthermore, a significant number of cells leads to high secretion of metabolites, chemicals, and proteins, resulting in easy detection using standard analytic tools. Finally, our biochip integrating hydroscaffold was adapted to our OoC fluidic platform allowing middle throughput analysis (IDCCM [34]). 

## 5. Conclusions

In this study, we propose to create a relevant microenvironment for culturing liver cells. The technology relied on the combination of a hydroscaffold embedded inside a microfluidic device. This combination made it possible to perform the liver HepG2/C3A cell culture in a complex 3D dynamic configuration. The HepG2/C3A formed spheroids and then large clusters of spheroids in the whole biochip. The live/dead staining revealed a high viability, with weak necrotic tissue at the center of the spheroids. Furthermore, tissue polarity was demonstrated by the MRP2 and BSEP networks, illustrating ongoing bile-like canicular network formation. The functional analysis demonstrated higher levels of albumin and urea secretions in the 3D cultures within the dynamic hydroscaffold-biochip conditions, when compared to the 3D hydroscaffold Petri controls. These results show the potential of combining organ-on-chip technology and hydroscaffold mimicking ECM to build relevant 3D liver models in vitro. We believe that the hydroscaffold-based liver-on-chip combined with primary hepatocytes or hiPSCs could play a role in producing a promising device for drug screening and risk assessment.

## Figures and Tables

**Table 1:** Albumin production (ng/h) for several seeded cell densities and culture modes.

	Seeded Cells	48 h	96 h
Biochip + hydroscaffold	1.25 × 10^5^ cells/cm^2^	66 ± 15	91 ± 18
	2.5 × 10^5^ cells/cm^2^	108 ± 13	132 ± 34
Petri + hydroscaffold	1.25 × 10^5^ cells/cm^2^	20.5 ± 2.55	29.5 ± 5
	2.5 × 10^5^ cells/cm^2^	18 ± 1.8	26.5 ± 3.2
Biochip **	1.25 × 10^5^ cells/cm^2^	95 ± 5	90 ± 40
	2.5 × 10^5^ cells/cm^2^	118 ± 25	190 ± 85
Biochip + alginate cryogel *	2.5 × 10^5^ cells/cm^2^	88 ± 25	135 ± 60

* Boulais et al., 2021; ** Baudoin et al., 2012; hydroscaffold = BIOMIMESYS^®^ Liver.

## Data Availability

The data presented in this study are available on request from the corresponding authors.

## Supplementary Materials

The following supporting information can be downloaded at: https://www.mdpi.com/article/10.3390/bioengineering9090443/s1, Figure S1: (A) Soft lithography process used for biochip fabrication; (B) Biochip design and dimensions., Figure S2: (A) Different compartments of IDCCM device; (B) IDCCM device (with biochip in the bottom) connected to a peristaltic pump; (C) Principle of the IDCCM device and perfusion cultures., Figure S3: Setup used for pressure measurement, Figure S4: Morphology of spheroids in well-plate containing hydroscaffold after 96 h of culture: (A) low, (B) intermediate and (C) high starting densities., Figure S5: DAPI (nuclei), calcein (living cells), and ethidium (dead cells) staining of spheroids after 21 days of culture in a biochip containing a hydroscaffold, Figure S6: DAPI (nuclei), phalloidin (F-actin) and E-cadherin staining of spheroids after 21 days of culture in a static well-plate containing a hydroscaffold, Figure S7: SEM images of cell spheroids cultured 21 days in a biochip containing a hydroscaffold, Figure S8: Albumin secreted by HepG2/C3A cells in 2D and 3D (hydroscaffold) static cultures and Figure S9: Morphologies and F-actin staining of HepG2/C3A inside a PDMS biochip (without hydrogel/hydroscaffold).
