# Supplementary material for: Development of Liver-on-Chip Integrating a Hydroscaffold Mimicking the Liver’s Extracellular Matrix
Source: Bioengineering (Basel). 2022 Sep 5;9(9):443. doi: 10.3390/bioengineering9090443 (PMC9495334; doi:10.3390/bioengineering9090443)
Supplement: Supplementary file 1 [file bioengineering-09-00443-s001.zip › bioengineering-1857809-supplementary.pdf]

bioengineering

Article

Development of Liver-On-Chip Integrating a Hydroscaffold Mimicking the Liver's Extracellular Matrix

Taha Messelmani [1], Anne Le Goff [1,*], Zied Souguir [2], Victoria Maes [1,2], Méryl Roudaut [2], Elodie Vandenhaute [2], Nathalie Maubon [2], Cécile Legallais [1], Eric Leclerc [1,3] and Rachid Jellali [1,*]

[1] CNRS, Biomechanics and Bioengineering, Centre de Recherche Royallieu-CS 60319, Université de Technologie de Compiègne,60203 Compiègne, France; taha.messelmani@utc.fr (T.M.); vic.maes@yahoo.fr (V.M.); cecile.legallais@utc.fr (C.L.); eleclerc@iis.u-tokyo.ac.jp (E.L.)
[2] HCS Pharma, 250 rue Salvador Allende, Biocentre Fleming Bâtiment A, 59120 Loos, France; zied.souguir@hcs-pharma.com (Z.S.); meryl.roudaut@hcs-pharma.com (M.R.); elodie.vandenhaute@hcs-pharma.com (E.V.); nathalie.maubon@hcs-pharma.com (N.M.)
[3] CNRS IRL 2820, Laboratory for Integrated Micro Mechatronic Systems, Institute of Industrial Science, University of Tokyo, 4-6-1 Komaba, Meguro-ku, Tokyo 153-8505, Japan
* Correspondence: anne.le-goff@utc.fr (A.L.G.); rachid.jellali@utc.fr (R.J.)

Supplementary figures

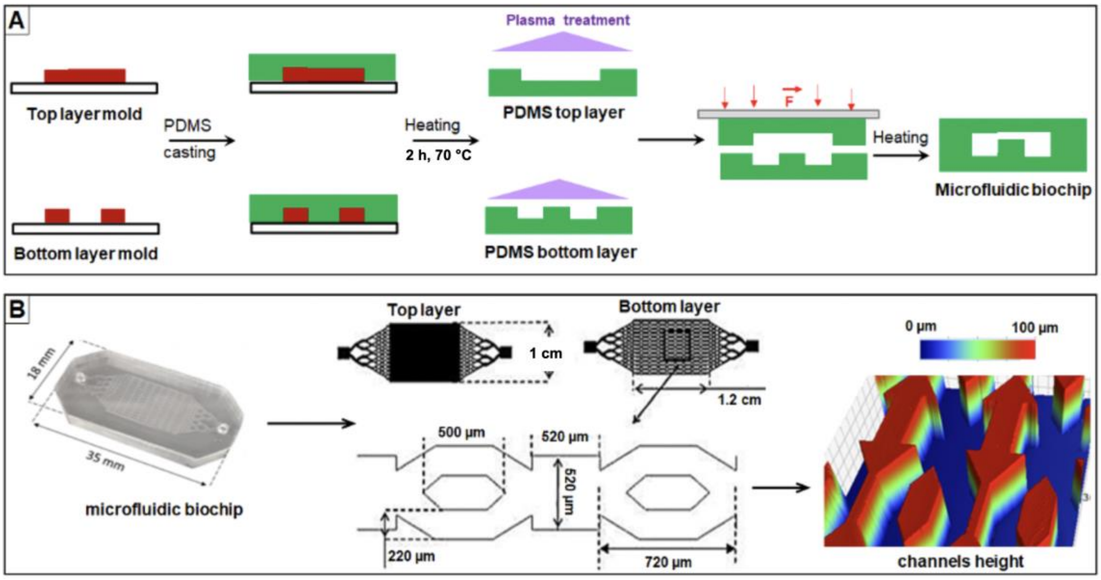

Figure S1. (A) Soft lithography process used for biochip fabrication; (B) Biochip design and dimensions.

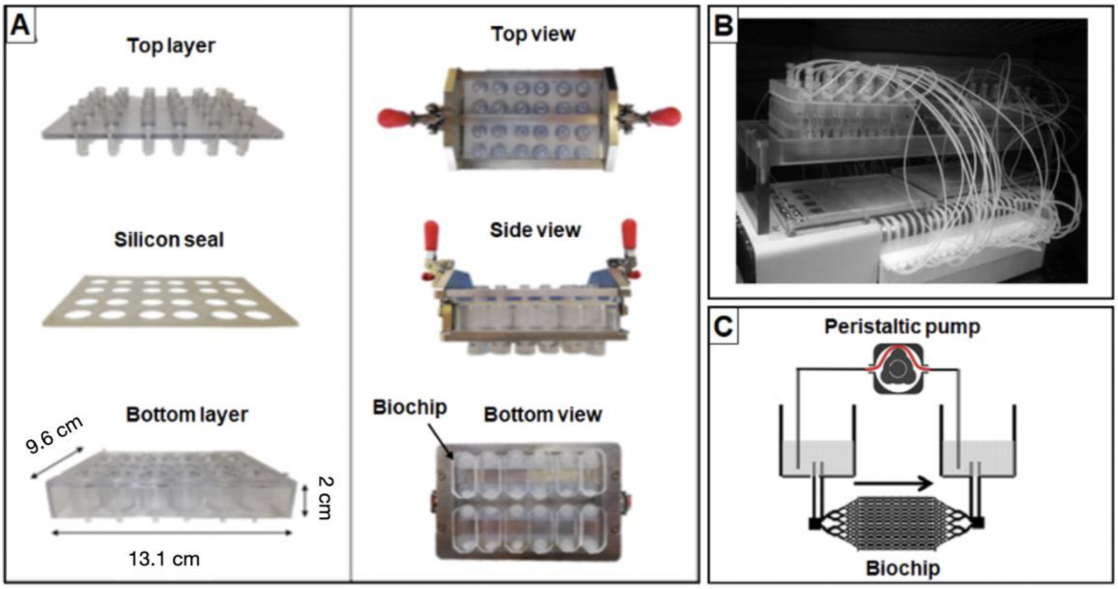

Figure S2. (A) Different compartments of IDCCM device; (B): IDCCM device (with biochip in the bottom) connected to peristaltic pump; (C) principle of the IDCCM device and perfusion cultures.

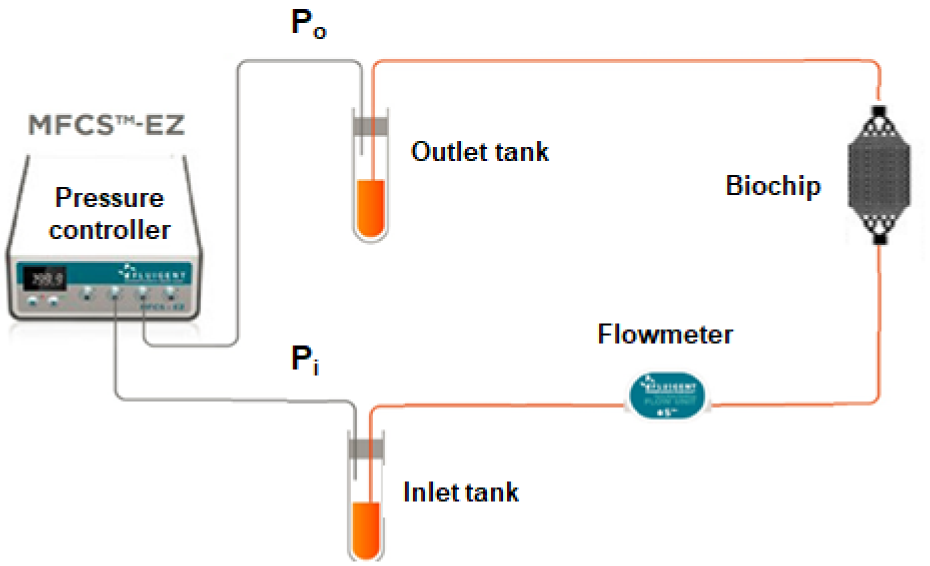

Figure S3. Setup used for pressure measurement.

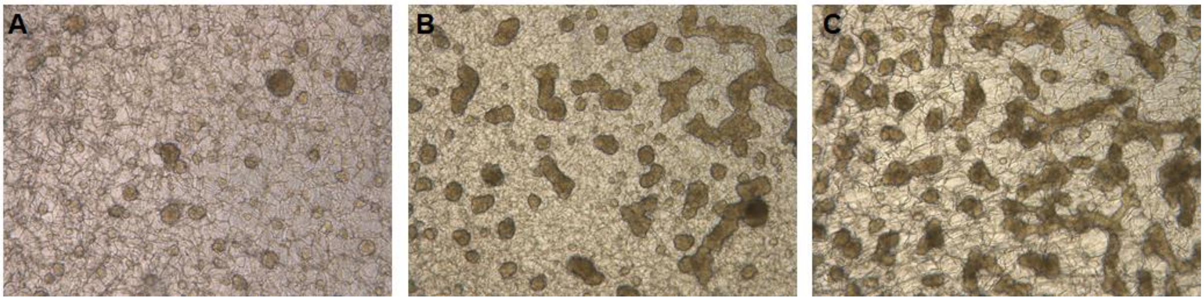

Figure S4. Morphology of spheroids in well-plate containing hydroscaffold after 96 h of culture: (A) low, (B) intermediate and (C) high starting densities.

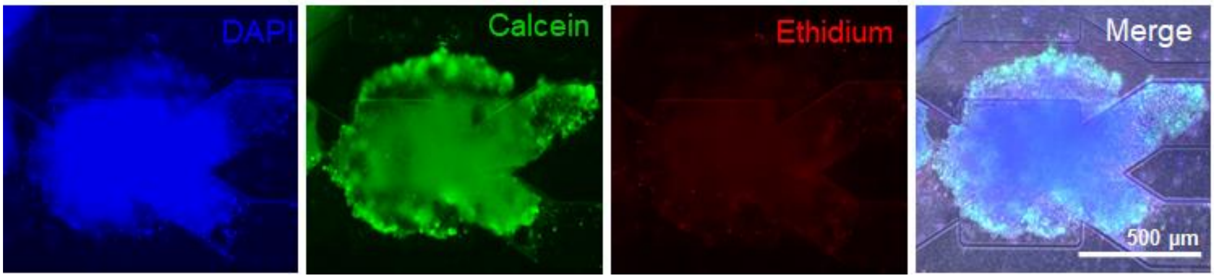

Figure S5. DAPI (nuclei), calcein (living cells) and ethidium (dead cells) staining of spheroids after 21 days of culture in a biochip containing a hydroscaffold.

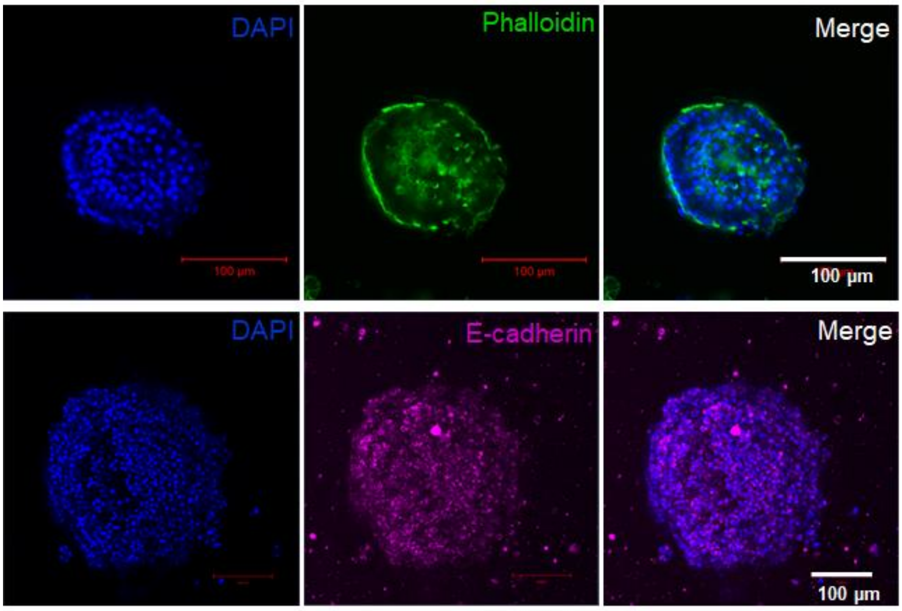

Figure S6. DAPI (nuclei), phalloidin (F-actin) and E-cadherin staining of spheroids after 21 days of culture in a static well-plate containing a hydroscaffold.

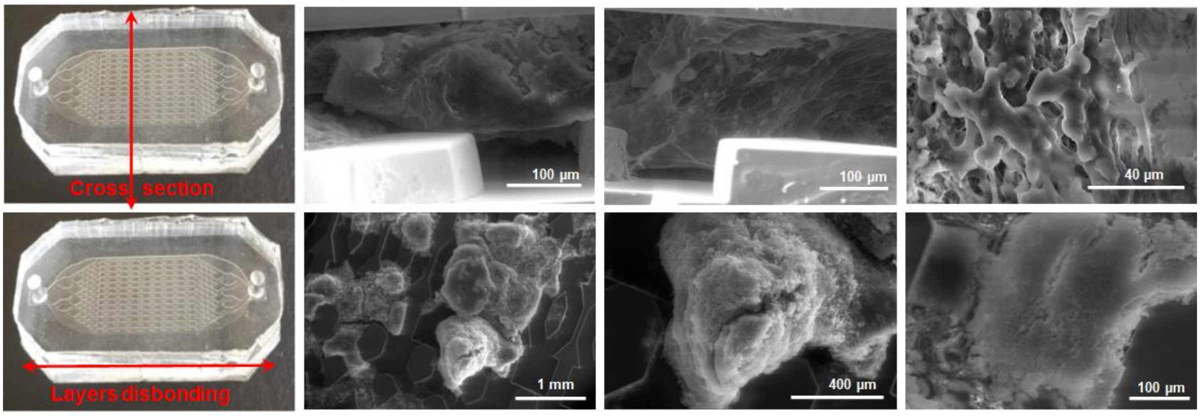

Figure S7. SEM images of cell spheroids cultured 21 days in a biochip containing a hydroscaffold.

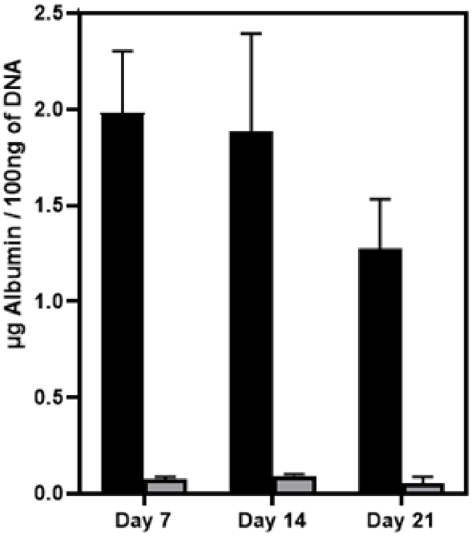

Figure S8. Albumin secreted by HepG2/C3A cells in 2D and 3D (hydroscaffold) static culture.

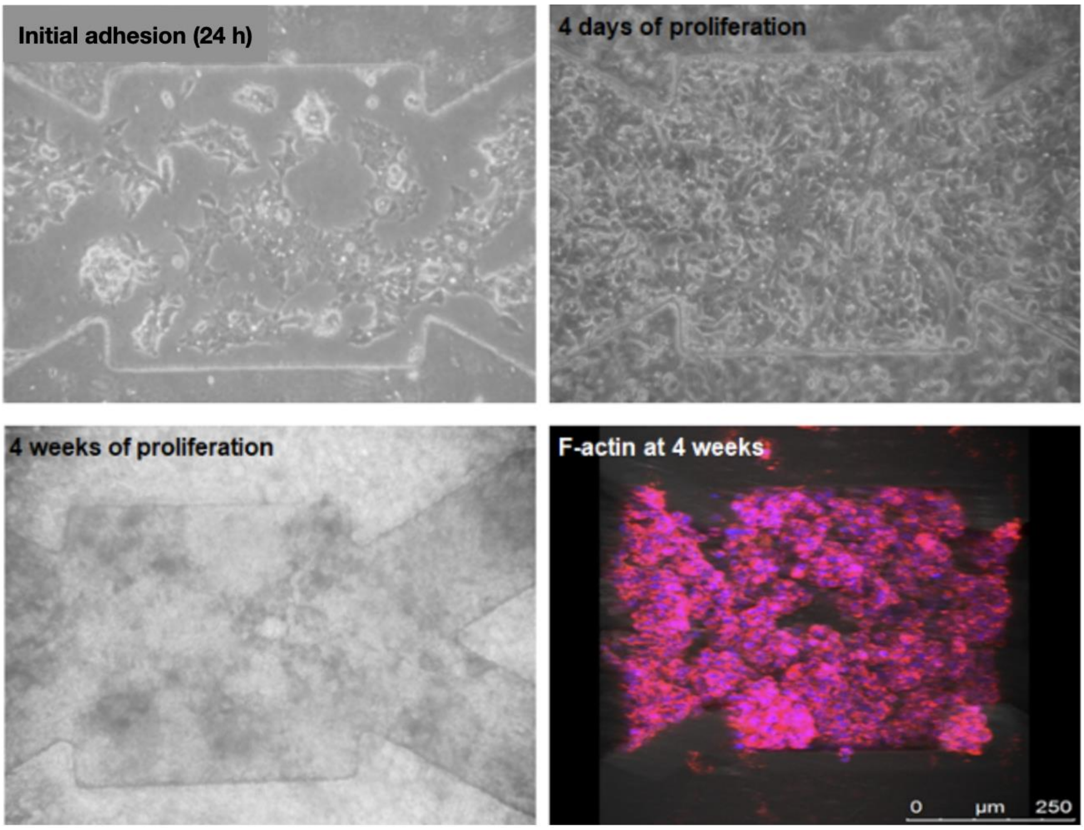

Figure S9. Morphologies and F-actin staining of HepG2/C3A inside a PDMS biochip (without hydrogel/hydroscaffold).
